# LEAP2 in Physiology—A Narrative Review

**DOI:** 10.3390/ijms26010377

**Published:** 2025-01-04

**Authors:** Oskar Sosinski, Ewa Pruszynska-Oszmalek, Natalia Leciejewska, Maciej Sassek, Pawel Antoni Kolodziejski

**Affiliations:** Department of Animal Physiology, Biochemistry and Biostructure, Poznan University of Life Sciences, Wolynska 35 Street, 60-637 Poznan, Poland; oskar.sosinski@up.poznan.pl (O.S.); natalia.leciejewska@up.poznan.pl (N.L.); maciej.sassek@up.poznan.pl (M.S.)

**Keywords:** Liver Enriched Antimicrobial Peptide 2 (LEAP2), GHSR, metabolism, mammals, avian, fish, reptiles, amphibians

## Abstract

Liver Enriched Antimicrobial Peptide 2 (LEAP2) is a fascinating peptide that has gained significant attention since its discovery in 2003. Initially identified as an antimicrobial peptide, LEAP2 has more recently been found to play a key role in the regulation of energy metabolism. One of the most notable functions of LEAP2 is its interaction with the ghrelin hormone, which is known for stimulating hunger. LEAP2 acts as an inhibitor of ghrelin, thereby reducing food intake and influencing energy balance. The physiological roles of LEAP2 extend beyond appetite suppression. Studies have shown that LEAP2 has an impact on insulin secretion, suggesting its potential involvement in glucose metabolism and possibly insulin sensitivity, which is crucial in managing conditions like type 2 diabetes. Moreover, LEAP2 levels appear to fluctuate based on factors such as gender, developmental stage, and even interventions like bariatric surgery, which is known for its role in managing obesity and diabetes. Given these findings, LEAP2 shows potential as a therapeutic target, particularly for addressing obesity and metabolic diseases such as type 2 diabetes. Its ability to influence food intake and energy balance makes it a promising candidate for further research into therapies aimed at weight regulation and glycemic control. In the future, LEAP2 could become an important agent in the development of treatments aimed at curbing obesity and its associated metabolic disorders.

## 1. Introduction

This review focuses on the systematization of knowledge regarding the role of LEAP2 in the metabolism of animals and humans, as well as on a critical approach to the currently obtained results and the identification of potential directions for future research on this peptide.

LEAP2 was first identified in human blood ultrafiltrate in 2003 by Krause [1], and subsequent studies discovered its presence in *Gallus gallus* (chicken) [2] and *Oncorhynchus mykiss* (rainbow trout) [3]. In the first article about LEAP2 [1], the acronym stood for “Liver Expressed Antimicrobial Peptide 2” While this name has occasionally appeared in more recent publications, the correct term, as designated by the HUGO Gene Nomenclature Committee (HGNC), is “Liver Enriched Antimicrobial Peptide 2”. Additionally, the acronym LEAP2 is sometimes used interchangeably with LEAP-2, both of which are considered correct.

**Figure 1:**
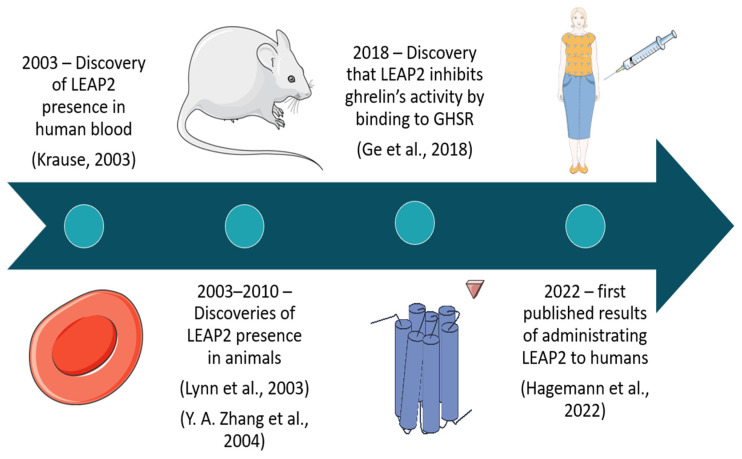
Key dates regarding the discovery and role of LEAP 2 (based on [1,2,3,7,10]). Picture source: https://smart.servier.com/, accessed on 3 March 2024.

Initially, LEAP2 was classified as an antimicrobial peptide due to its elevated plasma levels during illness, as observed by Francisco et al. and Sakai et al. [4,5]. This hypothesis was further supported by research on fish, where *LEAP2* expression increased in internal organs and skin after *Vibrio anguillarum* infection in *Cyprinus carpio* [6]. These findings suggested a role for LEAP2 in immune defense, both in the bloodstream and at mucosal barriers, particularly in the skin of fish. However, a key breakthrough in LEAP2’s physiological role came in 2018 when it was discovered to function as an antagonist of Growth Hormone Secretagogue Receptor (GHSR). LEAP2 inhibits GHSR by blocking ghrelin, the receptor’s natural ligand, which plays a critical role in hunger signaling. The IC50 (half-maximal inhibitory concentration) of LEAP2 for inhibiting GHSR activation was found to be 6 nM while its physiological plasma concentration was around 2 nM [7]. This contrasted sharply with the IC50 for LEAP2’s antimicrobial activity, which was much higher at approximately 5 µM [1]. A difference of three orders of magnitude in IC50 implies that LEAP2’s primary role is more likely related to metabolic regulation than to antimicrobial defense. LEAP2 is not unique in this respect. Another peptide, hepcidin (formerly known as LEAP-1), was initially classified as an antimicrobial peptide before its critical role in iron homeostasis was uncovered [8]. While research has also considered whether LEAP2 is involved in other physiological processes such as iron regulation or hepatocyte proliferation, no links have been established [9] These insights position LEAP2 primarily as a regulator of metabolism, particularly in the context of ghrelin signaling, rather than as a key player in immune defense (Figure 1).

## 2. Structure of LEAP2

LEAP2 exists in several forms circulating in the blood, differing in the amino acid sequence length but sharing a core structure formed by two disulfide bonds, created by cysteine residues positioned in the relative 1–3 and 2–4 positions [1]. This core structure includes key elements such as a β-hairpin and a 3_10_-helix, which contribute to the peptide’s stability and function [11]. In humans, the LEAP2 gene is located on chromosome 5 (5q31.1) and comprises three exons and two introns. The mRNA codes the precursor protein made up of 77 amino acid residues. Interestingly, the mRNA precursor undergoes alternative splicing, producing multiple forms of mRNA. These spliced variants include forms that retain the first intron, the second intron, or both [1]. These products of alternative splicing might be located in various tissues together with fully spliced mRNA sequences, with the exception of the liver, where only fully spliced mRNA has been detected. An alternatively spliced *LEAP2* sequence that kept one intron might be missing a signal aa sequence, with suggests an intracellular destination, while a fully spliced LEAP2 protein has an extracellular destination. This suggests that LEAP2 undergoes alternative splicing in various tissues to produce distinct yet familiar peptides [9].

The human LEAP2 preprotein weighs approximately 8.814 kDa and consists of 77 amino acid residues. After undergoing post-translational modification, the largest mature form of LEAP2 consists of residues 38–77, with a molecular weight of 4.585 kDa. The initial 22 amino acids (residues 1–22) function as a signal peptide and residues 23–37 are cleaved off during maturation. The resulting mature LEAP2 peptide is made up of residues 38–77. Disulfide bonds are formed between residues 54 to 65 and 60 to 70, stabilizing its structure. Other forms of LEAP2 can also circulate in the blood, including variants like 39–77, 43–77, 48–77, 48–73, 48–75, 46–76, and 48–76, indicating a level of structural flexibility. Interestingly, LEAP2 shares features common to antimicrobial peptides such as the presence of multiple positively charged amino acids, which facilitates interactions with Gram-positive bacteria. Additionally, LEAP2 has a predicted isoelectric point (pI) of 8.95 and contains a characteristic amino acid cluster, -RKRR-, further supporting its antimicrobial potential [1,3]. Secondary structures like β-sheet or α-helix have not been detected. The preprotein contains a furin cleavage site located ammino-terminally to Met38 [1]. The core of LEAP2 contains the majority of lysine and arginine residues and is stabilized by two disulfide bonds and hydrogen bonds [11].

The N-terminal region of LEAP2, particularly comprising residues 1–14, plays a crucial role in its interaction with GHSR. This has been demonstrated by research showing that LEAP2 lacking its N-terminal end (15-40-OH) does not inhibit ghrelin-induced food intake [12]. Within the N-terminal sequence, the first 1–8 amino acid residues contain key binding determinants while a longer segment of 1–12 residues is necessary to achieve the maximal binding efficiency of LEAP2 to the receptor [12]. Moreover, the N-terminal region’s hydrophobicity is essential for anchoring the protein to the phospholipid bilayer of the target cell membrane, which facilitates its interaction with GHSR. While both the N-terminal region and the core of LEAP2 are important for binding, the C-terminal end appears to be less critical. Studies have shown that truncated versions of LEAP2, which lack the C-terminal portion, can still bind effectively to the membrane, but the N-terminal region alone shows weaker binding [11]. The presence of a tryptophan residue (Trp) in the N-terminal region likely plays a significant role in membrane targeting as tryptophan is known to be a key determinant for membrane association [13]. This structural feature enhances LEAP2’s ability to interact with its target receptor, GHSR, and modulate its physiological effects.

Despite LEAP2_38–77_ being the most common type of LEAP2, other variants also have interesting properties: for example, LEAP2_38–47_ is detectable in human plasma and acts as an insulin secretagogue in human pancreas islets in vitro. Alas, it does not affect the insulin response in healthy humans in vivo. 

**Figure 2:**
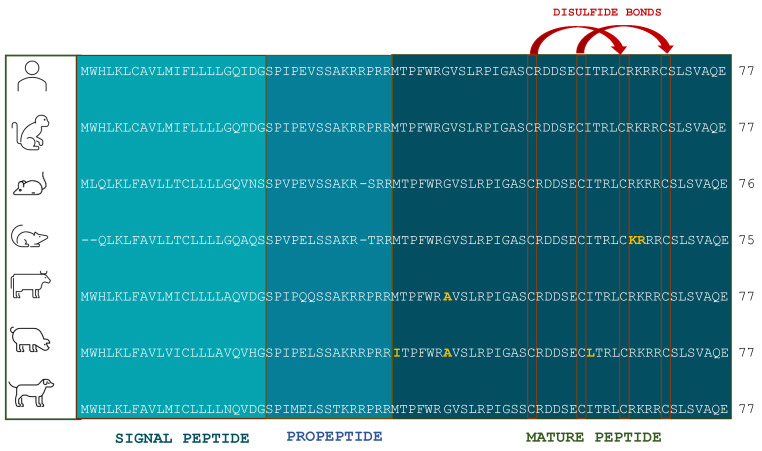
Comparison of the LEAP2 amino acid sequence in humans and other mammalian species. From top: human (*Homo sapiens*), monkey (*Macaca mulatta*), mouse (*Mus musculus*), rat (*Rattus norvegicus*), cattle (*Bos taurus*), pig (*Sus scrofa*), and dog (*Canis familiaris*). Yellow color shows differences in amino acid sequence between humans and chosen animals.

The structure of LEAP2 is remarkably conserved across mammals including species such as cows, mice, and Rhesus monkeys. These animals share the same total number of amino acids (40), net charge (+4), and predicted isoelectric point (pI) of 8.95 with the human version of the peptide. One of the key structural features is the presence of four cysteine residues, which are located in identical positions among these mammals and are responsible for forming disulfide bonds that stabilize the peptide’s structure. Additionally, an -RXXR- motif at the cleavage site is also conserved in various mammalian species. This cleavage site, which separates the prodomain from the mature region of LEAP2, remains highly conserved not only in mammals but also in fish, reflecting the evolutionary stability of this region [3]. Studies have further shown that LEAP2 exhibits nearly 100% homology in its amino acid sequences among mammals, underscoring its structural and functional conservation across species [9] (Figure 2).

## 3. The Antimicrobial Function of LEAP2

LEAP2 belongs to the cationic antimicrobial peptide (CAMP) family, a group of small proteins (typically less than 10 kDa in size) secreted by epithelial cells that act to destroy pathogens. These peptides typically kill microbes through various mechanisms such as modulating chemotaxis, inducing apoptosis, or altering gene transcription. CAMPs play a crucial role in the immune system through various mechanisms. Certain peptides, such as cathelicidins and defensins, directly kill bacteria by interacting with their membranes [14]. This interaction disrupts their membrane structures, creating ion-permeable channels and increasing membrane permeability, ultimately leading to bacterial cell death. In addition to their direct antimicrobial activity, CAMPs can indirectly combat microbes by activating the immune system. When secreted at infection sites, they stimulate histamine release and promote the secretion of chemokines and antiviral cytokines [14]. Peptides like α-defensins also exhibit chemotactic properties, attracting monocytes and T cells to infection sites. Those actions mobilize cells involved in cellular and humoral immune responses. Furthermore, CAMPs such as cathelicidins and defensins enhance immune function by inducing dendritic cell differentiation, modifying phagocytic receptors, and increasing cytokine secretion [14].

In the case of LEAP2, its antimicrobial activity involves disrupting bacterial cell membranes through pore formation. However, this activity requires LEAP2 concentrations above physiological levels to be effective [9,11]. Like other CAMPs, LEAP2’s positive charge facilitates its binding to the negatively charged phospholipid membranes of bacteria, allowing it to interact more effectively with bacterial membranes than with the neutral membranes of blood cells [11]. LEAP2’s lack of toxicity toward somatic cells allows it to selectively target bacterial membranes without harming blood cells, ensuring an effective immune response within the bloodstream.

Despite these typical CAMP properties, LEAP2 stands out for its highly conserved amino acid sequence, which is unusual among CAMPs as these proteins usually evolve rapidly in response to specific host–pathogen interactions. For example, defensins have adapted through gene duplication and exon shuffling to combat infections more efficiently. The conserved nature of LEAP2’s sequence suggests that it plays a more critical role in physiological processes beyond its antimicrobial function. Unlike other CAMPs, LEAP2 lacks chemotactic properties and does not induce the migration of monocytes, further differentiating it from typical antimicrobial peptides [9]. LEAP2 has shown antimicrobial activity against both Gram-positive bacteria (e.g., *Bacillus megaterium*, *Bacillus subtilis*) and Gram-negative bacteria (e.g., *Neisseria cinerea*), as well as against certain yeasts like *Saccharomyces cerevisiae* and *Rhodotorula muciloginosa* [1]. This highlights its broad-spectrum potential despite its primary role in metabolic regulation.

Although it was discovered that LEAP2 cannot affect bacteria at normal concentrations and its main role is interaction with GHSR and ghrelin [7], there have been discoveries of LEAP2 concentration levels being raised during sickness [4,5]. During bacterial meningitis, human cerebrospinal fluid shows an increase in LEAP2 concentration compared to control groups and groups suffering from other neurological diseases, and this concentration drops twofold after antibiotic treatment. Moreover, LEAP2 in patients shows a positive correlation with inflammatory parameters such as C-reactive protein (CRP) (r = 0.624), but not with the Body Mass Index (BMI) [4]. Another example is rheumatoid arthritis (RA) in humans. In this case, LEAP2 serum levels are higher in ill people compared to healthy groups. LEAP2 is also positively correlated with inflammatory parameters, e.g., Interleukin-6 (IL6) (r = 0.8531), Interleukin-8 (IL8) (r = 0.7011), CRP (r = 0.1835), and Lipocalin-2 (LCN2) (r = 0.5648), but not with BMI [5]. Results for CRP might require more research for patients with RA and other diseases connected to this marker. That is because while the *p*-value shows the result of statistical significance, the correlation between LEAP2 and CRP (r = 0.1835) in this paper is very weak. This shows that LEAP2 could be potentially used as a biomarker of diseases such as bacterial meningitis or rheumatoid arthritis. However, further research is recommended to explore the correlation between LEAP2 and markers of various diseases, supplementing the current understanding.

## 4. GHSR Pathway Mechanisms

GHSR is a protein highly expressed in the central nervous system. GHSR is a G-protein-coupled receptor (GPCR) that forms heterodimers with receptors such as dopamine 2 receptor (D2R), somatostatin receptor 5 (SSTR5), or serotonin receptor type 2C (5-HT2c receptor) [15,16]. Its ligands are ghrelin and LEAP2; however, if ghrelin is not present, GHSR shows ligand-independent activity [17]. Its primary function is to mediate growth hormone release by interaction with ghrelin; however, GHSR shows constitutive activity for modulating hunger, which comes from interactions with other GPCRs. This way, GHSR can control behaviors related to food such as binge eating or food-seeking behavior in both ghrelin-dependent and independent ways [18].

Ghrelin was initially identified as a growth hormone secretagogue predominantly produced in the stomach [19]. However, over time, researchers have discovered that its functions extend far beyond growth hormone regulation. Ghrelin plays a key role in regulating appetite, adiposity, fertility, memory, learning, and glucose metabolism. By interacting with the central nervous system, particularly through its receptor (GHSR) in the hypothalamus, ghrelin promotes increased food intake and body weight gains. One of ghrelin’s critical roles is in stimulating neurogenesis in the hippocampus, a brain region essential for memory and learning [20]. A reduction in hippocampal neurogenesis, which can occur in the absence of adequate ghrelin activity, has been linked to memory impairments and depressive-like behaviors. Additionally, this reduction is thought to play a role in the development of neurodegenerative diseases such as Alzheimer’s disease. Thus, ghrelin’s influence on the brain goes beyond appetite control, contributing to cognitive functions and emotional regulation [20,21].

Ghrelin is initially synthesized as proghrelin, a precursor protein that undergoes enzymatic processing to produce desacyl ghrelin (DAG) and obestatin [22,23]. The active form, acyl ghrelin, is generated when proghrelin is cleaved and acylated by the enzyme ghrelin O-acyl transferase (GOAT) [24]. Acyl ghrelin binds to GHSR and is responsible for stimulating growth hormone release, as well as for regulating hunger and energy metabolism.

Researchers have developed synthetic antagonists of GHSR such as [D-Lys3]-GHRP-6, a ghrelin analogue that stimulates growth hormone release and modulates the ghrelin-GHSR pathway [25]. Elevated plasma levels of ghrelin have been associated with metabolic diseases such as obesity, diabetes, and Prader–Willi syndrome [26,27]. The observed elevation of plasma ghrelin levels in metabolic diseases highlights its critical role in energy balance and appetite regulation. Conversely, patients with anorexia nervosa produce high levels of ghrelin but exhibit ghrelin insensitivity, which limits its appetite-stimulating effects [28]. This is an example of a compensatory mechanism in response to insensitivity, which aims at preventing further weight loss.

Obestatin, another product of proghrelin processing, was originally thought to have an unknown receptor, but more recent studies have suggested that it may interact with GHSR, possibly to stimulate insulin secretion in β-pancreatic cells under hyperglycemic conditions [29]. Obestatin appears to act as an antagonist to ghrelin, counteracting its effects on appetite, food intake, and growth hormone secretion [30,31]. Blocking GHSR also eliminates obestatin’s positive effects on cell survival, further supporting the antagonistic relationship between these two peptides [32].

When it comes to interaction between GHSR and LEAP2, it was believed that LEAP2 works as a non-competitive antagonist; however, the latest studies have shown that LEAP2’s relation to GHSR depends on the situation as LEAP2 can also function as an inverse agonist and competitive antagonist [12,33].

Early research has shown that LEAP2 is a non-competitive antagonist as it reduces the magnitude of ghrelin-mediated GHSR activation, which could not be overcome by an increased ghrelin concentration. At the same time, competitive antagonists such as D-Arg^1^ or D-Phe^5^ have increased the EC_50_ of ghrelin while they have had no effect on maximal GHSR activation [7]. However, it is important to highlight the limitations of this research. First, the slow dissociation of LEAP2 has not been accounted for, which could have led to inaccurate results as LEAP2 was incubated for 30 min prior to the addition of ghrelin in the cited study. Slow dissociation could reduce the number of available GHSRs, thus reducing the maximal activation effect and creating the illusion of non-competitive behavior. Second, the experiment was conducted without a ligand-binding assay, a standard procedure necessary to reliably distinguish between competitive and non-competitive binding. 

Newer research argues that LEAP2 is actually a competitive antagonist. One of the arguments is that LEAP2 and ghrelin share the same ligand binding spot on GHSR as they both displace the ghrelin–SmBiT tracer from its binding spot with the same speed, which was proved by ligand-binding assays [33]. It is important to note that a LEAP2 concentration of 100 nM is enough to fully displace ghrelin from GHSR and to antagonize ghrelin in a cellular model [12,33]. That is because a physiological concentration of ~5 nM LEAP2 is not enough to affect in vitro models; therefore, a hyperphysiological concentration of 100 nM is often used in research. Another example has shown that LEAP2 acts as a competitive antagonist of ghrelin in ghrelin-induced IP1 production by displacing labelled ghrelin from its binding site, which has resulted in increased EC_50_ in ghrelin without changes to its maximal effect for IP1 production in HEK293T cells [12]. The competitive antagonism of LEAP2 also affects pancreatic islets; the N-terminal end of LEAP2 (1-12-NH_2_) was able to block ghrelin-induced insulin level reduction [12]. Considering the limitations of the initial research that suggested LEAP2 as a non-competitive inhibitor, along with the more recent and accurate studies that have proposed otherwise, we conclude that LEAP2 is most likely a competitive antagonist.

Antagonism towards ghrelin is not the only role of LEAP2. It can also act as an inverse agonist by modulating GHSR constitutive activity in a ghrelin-independent manner. Data indicate that LEAP2, together with its N-terminal region, stabilizes the inactive conformation of GHSR. By stabilizing the inactive conformation of GHSR, LEAP2 affects ghrelin-dependent and ghrelin-independent allosteric interactions between GHSR and other G-protein-coupled receptors such as dopamine 2 receptor [34]. This conformation is dissociated from Gq protein; therefore, GHSR cannot modulate the effects of D2R signaling on presynaptic Ca_v_2.2 currents in neurons. This can affect dopamine reward behavior when it comes to the consumption of palatable food. Ref. [35] showed that administrating LEAP2 to the central nervous system attenuates dopamine reward mechanism activated after accessing palatable food; for example, the treated mice expressed much lower interest in foods such as peanut butter compared to the control group. The changes in food habits related to dopamine observed following LEAP2 administration further confirm the role of this hormone in appetite regulation. Another proof that LEAP2 acts as an inverse agonist shows that fasting human plasma LEAP2 concentration has an inverse association with hunger sensation independent of plasma ghrelin concentration. This suggests that LEAP2 can interact with hunger sensation mechanisms, such as dopamine reward behavior, without the need for interaction with ghrelin [36].

This section has shortly described the GHSR pathway because it is known as the main target for LEAP2. However, there are controversies around LEAP2’s potential receptors. While it is known that LEAP2 interacts with GHSR, there have been contradicting articles about the existence of other receptors that can interact with LEAP2. Ref. [37] showed that the continuous administration of LEAP2 to calorie-restricted GHSR knockout mice resulted in a reduced body mass compared to the control group. On the other hand, ref. [10] showed that the application of LEAP2 to GHSR knockout mice showed no changes in plasma glucose levels while wild-type mice showed a diminished plasma glucose rise during ad libitum food intake. It is possible that these results depend on factors such as the way of administrating LEAP2 or the phenotype of the tested organism.

## 5. The Influence of LEAP2 on Physiology

### 5.1. Fish Physiology

Although LEAP2 was discovered over 20 years ago and its role in mammals is becoming better understood, the physiological function of this peptide in lower vertebrates is not well known. The first of these groups of animals comprises fish. Studies on both the sequence and the physiological role of this peptide have shown that in this group of animals, there is great diversity in terms of both LEAP2 forms and the genes encoding this protein.

Research carried out so far has shown that in some species such as *Danio rerio* (zebrafish), *Ctenopharyngodon idella* (grass carp), and *Trachinotus blochii* (golden pompano), LEAP2 is encoded by one gene, while in others, such as *Larimichthys crocea* (yellow croaker), *Acipenser baerii* (Siberian sturgeon), *Oncorhynchus mykiss* (rainbow trout), and *Cyprinus carpio* (common carp), it is encoded by two—or even three, in the case of species such as *Glyptothorax zanaensis*, which give rise to more than one LEAP2 subtype, such as LEAP2A, LEAP2B, and LEAP2C [38,39,40,41].

Despite such diversity in LEAP2 subtypes in fish, most studies on the role of this peptide have indicated their involvement in antimicrobial response. Studies on the antimicrobial role of LEAP2 in fish have revealed that this peptide exhibits selective antimicrobial activity against *Escherichia coli*, *Aeromonas hydrophila*, *Staphylococcus aureus*, and *Streptococcus agalactiae*. It disrupts bacterial cell membrane integrity and strongly binds to bacterial genomic DNA [38,42]. These selective antimicrobial properties and mechanisms of action have also been demonstrated in mammals [11], indicating that the antimicrobial effects of LEAP2 have been conserved throughout evolution.

Fish LEAP2 also interacts with GHSR; research on *Latimeria chalumnae,* a close living relative of tetrapods, has shown that LEAP2 antagonizes GHSR activation caused by ghrelin. Interestingly, human LEAP2 and ghrelin are as efficient as their fish orthologs when interacting with fish GHSR. However, when the situation is reversed, fish peptides have lower activity towards human GHSR [43]. This shows that the LEAP2–ghrelin–GHSR relation was already present in ancient fish and evolved with time.

### 5.2. Amphibian and Reptilian Physiology

Research about the role of LEAP2 in amphibians and reptiles is not common; however, scientists have proven LEAP2’s influence on infections and the GHSR pathway [44,45,46,47].

In the case of amphibians, research on *Xenopus tropicalis* (Western clawed frog) has shown the expression of *LEAP2* mainly in the intestines and liver. Mature LEAP2 consists of 40 amino acids. Research has shown that human LEAP2 antagonizes ghrelin-induced GHSR activation in *Xenopus tropicalis*, which proves both LEAP2’s role in energy metabolism and high similarity between amphibian and human LEAP2 [43]. Antimicrobial properties have been well shown in the case of *Leptobrachium liui* (moustache toad). During infection, *LEAP2* expression is upregulated in the skin, and it disrupts the bacterial cell membrane and hydrolyzes bacterial gDNA [45].

In the case of reptiles, *LEAP2* expression has been shown in *Pelodiscus sinensis* (softshell turtle) and detected only in the liver. Precursor LEAP2 is composed of the 22aa signal sequence, 15aa prodomain, and 40aa mature protein. The mature protein shows ~70% sequence identity to the LEAP2 sequences of other tetrapods. The injection of the *Edwardsiella tarda* bacterium or STIV virus upregulates *LEAP2* expression in the liver [46].

Among all discussed animal classes, research on reptiles seems to be most limited: as of the time of writing this article, there have only been a few articles about the relationship between reptiles and LEAP2, and no research on reptilian LEAP2’s interaction with the GHSR pathway has been published. 

### 5.3. Avian Physiology

In the model organism for birds, *Gallus gallus*, the *LEAP2* gene is located on chromosome 13 and consists of three exons and two introns, encoding a protein that is 76 amino acids long. *LEAP2* in chickens is expressed in various tissues including the small intestine, kidney, lung, and liver. Interestingly, there are four splice variants of chicken LEAP2, which suggests that the peptide might perform different roles in various tissues [48]. *LEAP2* is highly conserved among avian species. For example, the exons of *Gallus gallus* and *Coturnix japonica* (Japanese quail) show over 90% nucleotide identity. Additionally, the amino acid sequence of mature LEAP2 is identical across species such as *Gallus gallus*, *Coturnix japonica*, *Meleagris gallopavo* (turkey), and *Charadrius vociferus* (killdeer), indicating that LEAP2 plays a critical role across different avian species [49]. The high level of similarity in nucleotide identity and amino acid sequence demonstrates that the *LEAP2* gene is highly conserved not only among mammals [9] but also across avian species.

Similar to what is noted in other species, avian LEAP2 exhibits antimicrobial properties, and research has focused on its role in diseases relevant to the poultry industry such as coccidiosis (caused by *Eimeria*) and salmonellosis (caused by *Salmonella*). There is a correlation between *LEAP2* expression and microbial infection. For instance, during Salmonella infection, *LEAP2* expression is upregulated in embryos and gonads, suggesting its role in preventing egg contamination [48,50]. In the case of *Salmonella*, it has been shown that chicken *LEAP2* expression in embryos and gonads is upregulated by *Salmonella* infection. This provides evidence for LEAP2’s important role in the prevention of egg contamination [51].

Research on mammal cells has proven the selectivity of LEAP2’s antimicrobial activity, [52] and this selectivity might apply for probiotics used in poultry farming because despite its antimicrobial properties, *LEAP2’s* expression does not seem to be affected by the application of *Lactobacillus*-based probiotics as there is no change in expression levels according to [53].

Inspired by mammalian LEAP2, chicken LEAP2 has been tested for relation to food intake. It has been shown that *LEAP2* expression levels are lower in the liver and small intestine in fasted chickens compared to fed chickens and that *LEAP2* expression in fasted chickens increased after feeding [54]. These findings suggest that avian LEAP2 plays dual roles in both immune defense and energy regulation, making it a critical protein in avian physiology, although further studies are required to fully describe the role of LEAP2 in avian metabolism.

### 5.4. Human and Mammal Physiology

#### 5.4.1. Production and Regulation of LEAP2 in Plasma

As mentioned earlier, the first paper on LEAP2’s interaction with ghrelin and GHSR was published in 2018. It reported that LEAP2 completely blocks ghrelin from binding to GHSR, thereby influencing food intake, growth hormone release, and glucose homeostasis, especially during periods of caloric restriction [7]. The physiological effects of exogenous LEAP2 can be dependent on various factors such as the dose (a higher dosage equals a longer suppression of appetite) and body weight (obese rodents have shown lower glucose levels and higher reductions in food intake than lean models) while endogenous LEAP2 is affected by BMI (a higher BMI means a higher plasma LEAP2 rise after meals) [55,56]. On the other hand, it has been shown that the effects of exogenous LEAP2 are not affected by discomfort, anorexigenic agents (obestatin), and GH levels [55].

As LEAP2 is related to food intake, in a study, digestive system was analyzed for expression. Indeed, the highest *LEAP2* expression was found in jejunum while tissues like the duodenum, liver, and ileum exhibited 20% of jejunum’s expression level. Other tissues showed minimal expression [7]. However, this research focused solely on gene expression, overlooking the actual production of the LEAP2 protein by organs such as the liver and small intestine. It is important to note that gene expression does not directly correlate with protein production as post-transcriptional processes, such as RNA interference, can regulate the final quantity of the translated protein. In fact, another study identified the liver as the primary source of LEAP2 production based on the concentrations of LEAP2 found in the blood of the portal vein, hepatic vein, and abdominal aorta [57]. The article that described the discovery of LEAP2 also identified the liver as its primary source, noting that the production of mature LEAP2 is facilitated by furin, an enzyme that is abundant in the liver [1]. It should be mentioned that while LEAP2 is produced by cells such as hepatocytes or enterocytes, this production is not their main function and it is unknown whether there are specialized endocrine cells that focus on LEAP2 production. 

Factors such as nutrition, hormones, and weight loss also regulate the presence of LEAP2. The administration of glucose or fat in the form of corn oil and palmitate has been shown to elevate serum LEAP2 levels and enhance *LEAP2* expression in the liver [57]. Conversely, ghrelin administration appears to decrease LEAP2 levels in the serum and reduce its expression in the liver [57,58]. Weight loss also impacts LEAP2 levels; in a study involving mice, both diet-induced and surgery-induced weight loss resulted in lower serum LEAP2 levels and reduced *LEAP2* expression in the liver, duodenum, and jejunum [57]. In summary, these findings reinforce the general belief that LEAP2 plays a role in promoting appetite suppression and weight loss. The administration of energy sources, such as carbohydrates and fats, leads to an increase in plasma LEAP2 levels as a physiological response whereas weight loss results in a decrease in plasma LEAP2 levels. Furthermore, the reduction in LEAP2 levels following ghrelin administration reflects a natural response to the increased presence of this antagonistic hormone.

Fasting conditions substantially lower the level of circulating LEAP2 in response to increased energy deficit while food consumption increases LEAP2 postprandially, demonstrating that an organism adjusts LEAP2 plasma levels in response to energy changes, decreasing its concentration during hunger and increasing it following food intake, as a tool to regulate appetite. It is important to point out that even though LEAP2 might have positive effects on insulin release and controlling appetite, it might be also dangerous if administrated to energy-deprived organisms. This has been shown by an experiment on mice wherein some mice were subjugated to a caloric restriction (CR) diet as the control group and the others were treated with the adenovirus-associated virus gene for *LEAP2* expression and fed with a CR diet. Both groups lost weight, but after 8 days, the control group was healthy and active while the LEAP2 group was too lethargic to eat food and was euthanized for ethical reasons [7]. The lethargy observed in mice with LEAP2 overexpression, to the point of being unable to eat, highlights the need for caution. If LEAP2 is ever considered as a therapeutic agent, its use should be carefully supervised by a medical professional.

LEAP2 levels drop not only during fasting but also during exercise due to created energy deficiency, and changes in LEAP2 levels depend on the intensity of exercise. This allows the body to increase ghrelin hunger signaling in order to replenish ghrelin stores [59]. Physical exercises and low-calorie diets (LCDs) may reduce the level of fasting LEAP2. However, the administration of glucose mixed with an LCD causes glucose-stimulated LEAP2 to rise compared to LCD + exercise groups [60].

A fall in plasma LEAP2 levels during energy deficit has been proposed as a neuroendocrine signal that mediates an upregulation of GHSR actions [61]. GHSR activates neurons of the hypothalamic paraventricular nucleus (PVH^CRF^ neurons) that take part in metabolic response caused by food deprivation. Since LEAP2 can act as an inverse agonist and the activation of PVH^CRF^ neurons is ghrelin-independent at hypothalamic level, LEAP2 can inhibit neuron activation. Therefore, falls in LEAP2 levels caused by energy deficit can upregulate GHSR activity during hunger [61].

When it comes to energy surplus, there is a positive correlation for LEAP2 with food intake and fat mass; obese mice have almost twice as much circulating LEAP2 as lean mice and plasma LEAP2 continues to rise with increases in weight. On top of that, the LEAP2/acyl-ghrelin ratio can be three times higher in obese mice compared to control mice [56]. The author of [56] suggests that changes in the LEAP2/acyl-ghrelin ratio observed in obesity, specifically the rise in LEAP2 levels and the decrease in acyl ghrelin, may be key factors contributing to the development of ghrelin resistance in obese individuals. Oral glucose administration also causes rises in LEAP2 levels and the LEAP2/acyl-ghrelin molar ratio while type 1 diabetes mellitus (T1DM) causes rises in LEAP2 levels without changes in the LEAP2/ghrelin ratio. Just like mice, obese humans also show increases in plasma LEAP2 and the LEAP2/acyl-ghrelin molar ratio; therefore, LEAP2 also positively correlates with some metabolic parameters connected to obesity such as the BMI, HOMA-IR, VAT volume, and serum triglycerides [56]. A positive correlation between LEAP2 and BMI has been assessed for adults; however, the relation between body weight and LEAP2 in children is poorly understood. There has been a report that BMI correlates negatively with circulating LEAP2 and there is no difference between obese and healthy children when it comes to the ghrelin/LEAP2 molar ratio, unlike in adults [62].

#### 5.4.2. Relation Between LEAP2 and Gender

Different researchers have stated that the LEAP2 level differs depending on age and gender. Girls have higher levels of circulating LEAP2 in both lean and obese examples than boys [63]. There is a statistically significant increase in plasma LEAP2 in girls who have reached puberty compared to prepubescent girls. For comparison, there is no increase in LEAP2 levels among maturing boys. The sexual dimorphism of LEAP2, especially during female puberty, suggests the role of LEAP2 in changes that occur during this time such as in energy balance, growth rates, and food intake. One possible explanation is that a raised LEAP2 level tones down the levels of the growth hormone elevated during puberty [63]. Another study has shown that circulating LEAP2 levels actually drop during male puberty [64], most likely to meet the increased demand for energy caused by increased growth rates and anabolic changes in organisms. The level of circulating LEAP2 correlates negatively with the HDL level and positively with the insulin level, HOMA-IR, and triglycerides (TGs), suggesting that a high level of LEAP2 in an adolescent boy might be a marker of an unhealthy metabolism of glucose and lipids [64].

Besides puberty, another interesting period in human life is pregnancy as it was discovered that LEAP2 takes part in pregnancy regulation [65]. The ghrelin/LEAP2 ratio reaches its peak during the second trimester of pregnancy in humans and rats. This might be associated with weight gain during pregnancy, as the anabolic phase of pregnancy lasts from the first to the second trimester of pregnancy, and it is associated with fat accumulation. Research suggests that during pregnancy, the production of LEAP2 is reduced, probably to favor metabolic effects of ghrelin on GHSR [65]. Preeclamptic pregnant women have shown no differences in the ghrelin/LEAP2 ratio with healthy pregnant women. This means that the ghrelin/LEAP2 ratio remains the same for both preeclamptic pregnant women and healthy pregnant women. Nevertheless, an important question remains: how are LEAP2 and ghrelin related to pregnancy disorders other than preeclampsia, such as miscarriage, gestational diabetes, or stillbirth? Can the ghrelin/LEAP2 ratio be used as a biomarker to monitor the status of pregnancy? Research on the relationship between LEAP2 and pregnancy is still scarce, with no specific studies investigating the interaction between the ghrelin/LEAP2 ratio and LEAP2 plasma levels in these conditions; therefore, this subject requires more future research.

#### 5.4.3. First Trial on Humans

Measurements of LEAP2 in people of different ages, genders, and body masses were followed by a trial with LEAP2 application. The first report about testing LEAP2’s administration on humans was published in 2022 [10]. This work showed that LEAP2 reduces postprandial plasma glucose levels. The infusion of LEAP2 in fasting conditions showed increases in plasma insulin levels and decreases in glycerol levels compared to the placebo group, suggesting insulinotropic and anti-lipolytic activity [10]. Exogenous LEAP2 does not change the level of plasma ghrelin, but it does lower the amount of released GH. In summary, the first human trial of exogenous LEAP2 confirmed several properties previously observed in animal studies, including appetite suppression, enhanced insulin secretion, and reduced postprandial plasma glucose levels. These findings validate the use of animal models for testing LEAP2’s effects. Patients who received LEAP2 showed lower appetites compared to the control. It should be pointed out that all patients were men; thus, it is unknown whether changes caused by LEAP2 infusion are gender-dependent [10]. It is recommended to conduct gender-specific research on women as studies have shown that LEAP2 plasma levels may be decreased in women with polycystic ovary syndrome (PCOS) [66]. This suggests that LEAP2 could have potential not only as a therapeutic agent for obesity and diabetes but also in the treatment of PCOS or as a biomarker for this condition.

#### 5.4.4. Appetite Regulation

The lower appetite after a human trial can be explained by ghrelin’s properties. While ghrelin interacts with GHSR to depolarize hypothalamic NPY neurons and stimulate appetite, the application of LEAP2 hyperpolarizes them, thus making them more resistant to stimulation. Furthermore, the application of LEAP2 can reverse ghrelin-stimulated membrane depolarization [56]. Since LEAP2 prevents the activity of ghrelin, it also indirectly lowers GH release and thus food intake. In an experiment with mice, a mouse treated with ghrelin showed a much higher appetite than a mouse pre-treated with LEAP2 and then treated with ghrelin, which might have been caused by the slow dissociation of LEAP2 from its binding spot. To evaluate the effects of the absence of LEAP2 on organisms, mice were injected with antibodies targeting LEAP2, which raised the peak release of GH in fasting mice compared to the control group [7] due to increased ghrelin activity resulting from reduced levels of functional LEAP2 in plasma. Another article describes the creation of *LEAP2* knockout mice, which became more sensitive to the effects of ghrelin on food intake and growth hormone secretion [67]. This means that the absence of LEAP2 promotes and even exaggerates ghrelin-mediated GHSR activity [7,67]. It is worth mentioning that *LEAP2* knockout mice showed no changes in body weight, food intake, or blood glucose if fed a normal chow diet. However, knockout females on a high-fat diet did show an increased appetite and body weight gain [67], most likely due to the disruption of the appetite-regulating mechanism driven by LEAP2 secretion. While it has not been explained why male rats do not show the same changes as females, the increased sensitivity to ghrelin in response to LEAP2 absence shown by both articles suggests that LEAP2 takes part in ghrelin resistance.

Since LEAP2 regulates appetite, which is controlled by the hypothalamus, researching how LEAP2 affects the central nervous system is the natural course of action. LEAP2 interacts with neurons in the arcuate nucleus of the hypothalamus (ARH) [68]. The delivery of the *LEAP2* gene into the ARH in the form of the adeno-associated virus (AAV) causes the overexpression of *LEAP2* in the hypothalamus and results in hypophagia and weight loss in mice fed on chow diets, but without changes to lean mass. What is more, the regular administration of ghrelin affects neither food intake nor body weight in the case of AAV mice, suggesting that the overexpression of *LEAP2* overrides ghrelin action [68]. Most importantly, *LEAP2* expression in ARH lowers body mass gain and food intake in the case of mice eating high-fat diets (HFDs), suggesting that LEAP2 may influence the hypothalamus’s regulation of hunger and satiety. The fact that both the chow diet group and HFD group have shown reductions in body mass suggests that the overexpression of *LEAP2* in the hypothalamus is calorie-independent [68].

#### 5.4.5. Insulin Secretion Regulation

The anorectic properties of LEAP2 might be very helpful in type 2 diabetes as this hormone might also affect insulin production. While it does not affect insulin secretion in ex vivo rat pancreatic islets stimulated by high glucose concentrations, it does negate ghrelin’s insulinostatic functions, thereby indirectly promoting insulin secretion. In a study, islets incubated with high levels of glucose produced less insulin if incubated with ghrelin, and islets incubated with both LEAP2 and ghrelin showed no reductions in insulin production. This shows that LEAP2 binds to pancreatic GHSR and functions as antagonist of ghrelin [69]. Another study proved that LEAP2 is associated with increased insulin secretion in adults with obesity and overweight [70]. Here, the LEAP2 plasma level was positively correlated with total body fat and insulin secretion. This provides insights into LEAP2 being used as a therapeutic agent for insulin secretion in type 2 diabetes and obesity as LEAP2 most likely directly increases insulin production rather than insulin sensitivity [70]. This role in insulin production might be helpful for people with type 2 diabetes. The authors of ref. [71] have discovered that in the case of humans, ghrelin levels are lowered and LEAP2 levels are increased in patients suffering from type 2 diabetes compared to control groups. This results in lowered ghrelin/LEAP2 ratios. This might be an organism’s attempt at blocking GHSR’s activity in order to return to proper energy homeostasis. In a study, LEAP2 was positively correlated with HbA1c parameter, with suggests LEAP2’s association with type 2 diabetes [71].

#### 5.4.6. Relation Between LEAP2 and RYGB Surgeries

*LEAP2* expression can be affected by bariatric surgeries such as Roux-en-Y gastric bypass (RYGB) or vertical sleeve gastrectomy (VSG). Articles such as [7,10] have shown that RYGB, also known as one of the most efficient obesity and type 2 diabetes treatments, lowers the level of plasma LEAP2. In the case of humans, lowered postprandial LEAP2 can be noticed after only 3 months while reductions in fasted LEAP2 can be detected after 2 years [56]. The results of VSG can be noticed 12 to 18 months after surgery, which indicates a decrease in postprandial LEAP2 release [56]. In summary, bariatric surgeries may reduce plasma LEAP2 levels; however, this effect is more likely attributed to the weight loss following the procedure rather than to the surgery itself as these changes often become apparent only after several months or even years.

#### 5.4.7. Relation Between LEAP2 and Diet

While LEAP2 is connected with food intake, the type of diet might affect LEAP2 levels. For example, it was revealed in a study that exposure to a ketogenic diet decreases LEAP2 levels in mice, probably due to presence of ketone body beta-hydroxybutyrate (BHB) created during fatty acid oxidation stimulated by this diet [59]. It was verified as the oral administration of BHB raised its plasma levels and reduced both the expression of *LEAP2* in the liver and duodenum and plasma LEAP2 levels. This confirms that a ketogenic diet downregulates LEAP2 expression. Since BHB is abundant in the livers of fasting organism, it might have a physiological role in reversing energy deficit during fasting [59].

## 6. Conclusions

**Figure 3:**
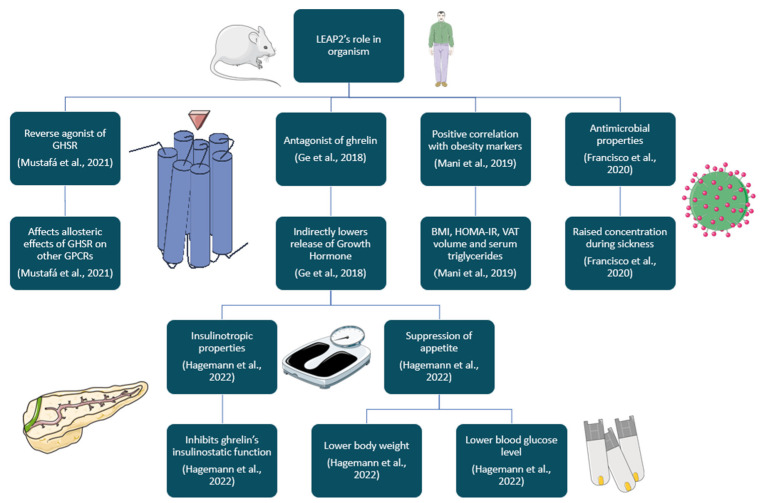
Summary of LEAP2 action in mammals (based on [5,7,10,34,56]). Picture source: https://smart.servier.com/, accessed on 3 March 2024.

**Figure 4:**
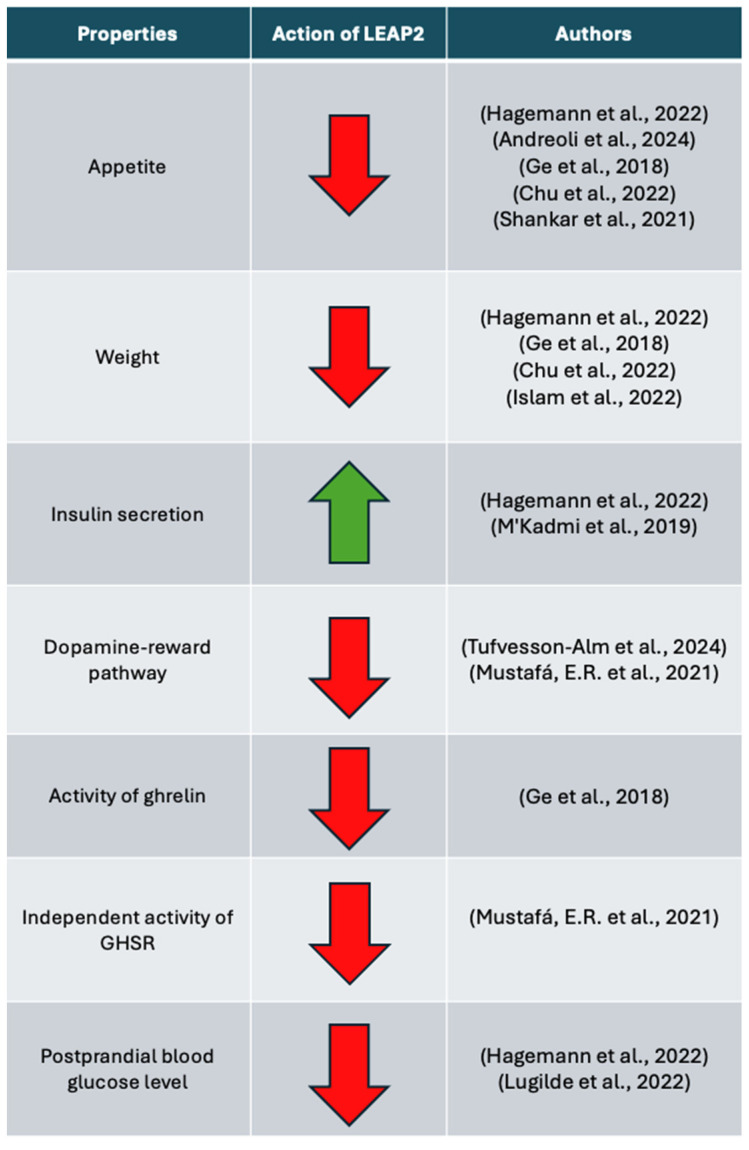
Summary of most important effects of LEAP2 on physiology (based on [7,10,12,34,35,36,37,55,67,68]). Red arrow down—downregulation of process; green arrow up—upregulation of process.

**Figure 5:**
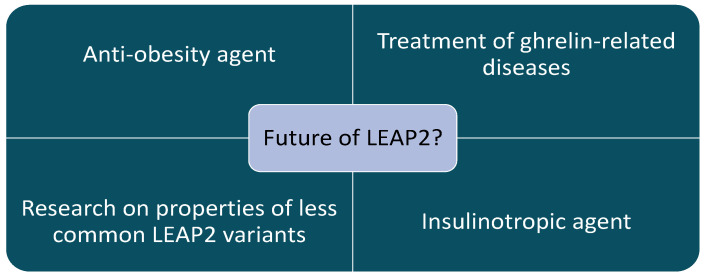
Anticipated future of research on LEAP2.

To conclude, the current state of knowledge pushes for the idea of LEAP2 having a benevolent influence on organisms suffering from diabetes and suffering from or developing obesity. However, there might be scenarios where LEAP2 shows no effect or even negative effects, such as lowering appetite during energy deficit or making contributions to the development of neurodegenerative diseases, due to the inhibition of ghrelin properties (Figure 3 and Figure 4). That is why it is important to continue research on animal models to dispel any doubts about LEAP2 properties, before conducing drug tests on humans. It might be also a good idea to study other variants of LEAP2, such as LEAP2_38-47_, because other variants might have different roles in organisms, which could prove useful in science and medicine (Figure 5).

For future research, we recommend focusing on type 2 diabetes. The first study on the administration of LEAP2 in healthy individuals was published in 2022, and in vivo research involving diabetic animal models is still limited. The next logical step would be to conduct more experiments using diabetes models and to use these findings as a key argument for the necessity of testing LEAP2 as a therapeutic agent for diabetic patients. As of now, there have been no published studies on the use of LEAP2 in treating diabetes patients. Additionally, further research should explore different animal classes as the majority of current studies have focused on mammals. Existing research on other classes of animals has not yet fully addressed critical topics such as the relationship between LEAP2, nutrition, body mass, energy changes, and diabetes.

## 7. Methods 

### Inclusion and Exclusion Criteria

This review has focused on papers examining LEAP2 and its interaction with animal physiology and GHSR pathways. Keywords used during the search included “LEAP2”, “LEAP-2”, “Liver Enriched Antimicrobial Peptide 2”, “diabetes”, “obesity”, “GHSR”, “antimicrobial activity”, “mammals”, “avians”, “reptiles”, “amphibians”, and “expression”. Information was sourced from reliable databases such as PubMed, Scopus, ResearchGate, Elsevier, and Google Scholar. No restrictions were applied regarding the publication date as LEAP2 was discovered in 2003 and all research since then remains relevant. Only papers written in English were included. Data that did not undergo scientific peer review such as abstracts, letters, and comments were excluded. Emphasis was placed on original research articles, with papers featuring questionable methodology, such as insufficient control groups, being omitted. Studies using human volunteers, animal cell lines, and in vivo animal models for research were prioritized.
